# De novo assembly and comparative analysis of the mitochondrial genome of *Reynoutria japonica*

**DOI:** 10.3389/fgene.2023.1289811

**Published:** 2023-11-23

**Authors:** Jianhui Chen, Hongping Ma, Haili Fan, Fan Lin, Tuanyao Chai, Hong Wang

**Affiliations:** College of Life Sciences, University of Chinese Academy of Sciences, Beijing, China

**Keywords:** *Reynoutria japonica*, mitogenome, codon usage, repeat sequences, RNA editing, evolution

## Abstract

*Reynoutria japonica* Houtt. is an important medical plant with a long history of thousands of years in China, however, its mitochondrial genome (mitogenome) has not been reported yet. In this work, we reported and analyzed the *R. japonica* mitogenome. The main results include: The *R. japonica* mitogenome was 302,229 bp in length and encoded 48 genes, including 27 protein-coding genes (PCGs), 3 rRNA genes, and 18 tRNA genes. Repeat sequence analysis revealed that there were 54 repeat sequences ranging from 193 bp to 1,983 bp in the *R. japonica* mitogenome. Relative synonymous codon usage (RSCU) analysis showed that leucine (900, 11.01%) and serine (732, 8.96%) were the two most abundant amino acids, and the codons with RSCU values showed the preference of A or T ending when greater than 1. The RNA editing sites of PCGs in the *R. japonica* mitogenome were characterized, and 299 RNA editing sites were found. Extensive sequences transfer between mitochondrion and chloroplast were found in *R. japonica*, where 11 complete plastid-derived tRNA genes stayed intact in the *R. japonica* mitogenome. Three genes (*ccmFC*, *cox1*, and *nad1*) were seen to play essential roles in the evolution through selection pressure analysis. The phylogenetic analysis showed that *Fallopia multiflora* was the closest species with *R. japonica*, in consistency with the results of chloroplast genome. Overall, the current work presents the first mitogenome of *R. japonica* and could contribute to the phylogenetic analysis of the family Polygonaceae.

## 1 Introduction

Plant mitochondria are semi-autonomous organelles, possessing relatively independent genetic systems and contributing to metabolism, energy production and cell homeostasis (Gualberto et al., 2014). It is generally believed that plant mitochondria were evolved from free-living bacteria according the endosymbiotic theory (Dyall et al., 2004). The mitochondria genome (mitogenome) in higher plants are very diversified in size, ranging from 22 Kb in *Avicennia marina* to 11.7 Mb in *Larix sibitia* (Putintseva et al., 2020; Friis et al., 2021) with distant genetic relationships, even between closely related species (Sloan et al., 2012; Cole et al., 2018). Due to the high frequency homologous recombination with foreign DNA, the mitogenomes in plants are often subject to rearrangement and more complex in size, structure and genes order (Wu et al., 2020). Note also that the homologous sequences in the seed plant mitogenomes are mainly derived from the chloroplast and nucleus (Drouin et al., 2008). Unlike chloroplast genome which is usually a double-stranded and circular molecule, plant mitogenome was found in multiple structural forms rather than the single ring form (Kozik et al., 2019). It is reported that many plant mitogenomes possess linear and branch structures, and a lot of smaller circular molecules (Sloan, 2013; Gualberto et al., 2014). For example, three loops were found in the mitogenome of *Hemerocallis citrina* and *Populus simonii* (Bi et al., 2022; Zhang et al., 2022). The repeat sequences are the main reasons that confused the ultimate conformation of mitogenome (Li et al., 2022; Yang et al., 2022). Overall, the plant mitogenome experienced sophisticated changes in size and structure during evolution, hence recovering the conformation of plant mitogenome is a both challenging and rewarding task.

*Reynoutria japonica* Houtt. (Polygonaceae), a well known traditional Chinese herbal medicine, has been used since ancient time in China (Peng et al., 2013). The dried root of *R. japonica* in combination with other traditional Chinese medicine herbs have multiple therapeutic uses (Zhang et al., 2013). In this ancient traditional Chinese medicine plant, the chloroplast genome of different regions *R. japonica* has been systematically analyzed (Chen et al., 2022). But its nuclear genome or mitogenome has not been published yet. Currently, the mitogenomes of the family Polygonaceae still remain largely unknown. Although the mitogenomes of *Fallopia multiflora* were assembled into two circular chromosomes via Illumina platform (Kim and Kim, 2018), few species mitogenomes in the family Polygonaceae are available in NCBI (National Center for Biotechnology Information) database. Therefore, it is necessary and desirable to attain the *R. japonica* mitogenome to enrich the Polygonaceae species mitogenome for further evolutionary studies. In recent years, an increasing interest of plant mitogenome is observed largely due to the advancement of sequencing technology and the reduction of sequencing costs, in particular, the application of Oxford Nanopore sequencing technology which has the advantage of long reading sequences to reduce the hassle caused by repetitive sequences compared to Illumina reads. Note that the combination of Illumina and Oxford Nanopore reads, several species mitogenome were obtained, including *Mesona chinensis* Benth (Tang et al., 2023), *Abelmoschus esculentus* (Li et al., 2022), *Hemerocallis citrina* (Zhang et al., 2022), *Photinia serratifolia* (Wang et al., 2023a), *etc*. These results demonstrate it is possible to assemble a complete mitogenome via the combination of short reads and long reads.

In this study, we sequenced and assembled the mitogenome of *R. japonica* via the Illumina short-read and Nanopore long-read integrated pipeline. The characteristic features of the *R. japonica* mitogenome were compared with those published related species. To our knowledge, this is the first assembly of the *R. japonica* mitogenome, which could be used for understanding the evolution of *R. japonica*, as well as the molecular biology research of this medicinal plant.

## 2 Materials and methods

### 2.1 Plant materials and genome sequencing

The seeds of *R. japonica* were collected from the medicinal plant garden of the Institute of Botany, Chinese Academy of Sciences (Beijing, China), planted and germinated in the lab and grown in a climate chamber at the temperature of 24°C ± 2°C with light/dark cycle of 16h/8 h. The well-grown young leaves were collected for DNA extraction. Total genomic DNA was isolated using the modified CTAB method (Arseneau et al., 2017). Then the quality of the extracted DNA was examined by NanoDrop (Thermo Scientific, United States), a Qubit fluorometer (Thermo Scientific, United States), and 0.75% agarose gel electrophoresis, respectively. The BluePippin system (Sage Science, United States) was used to recover large DNA fragments. Then the DNA fragments were treated using damage repair, end preparation, A-tailing, adapter ligation and the purification of DNA from the previous reaction using magnetic beads. The purified library was constructed following the SQK-LSK109 (Oxford, United Kingdom) sequencing kit protocol and loaded into a Nanopore GridION Sequencer (ONT, United Kingdom), which carried out at GrandOmics (Wuhan, China). Effective data were obtained by filtering adapter and removing low-quality reads. In total, 11.53 Gb of data were generated form 577,047 reads (SRA accession SRR24988768).

### 2.2 Genome assembly and annotation

The Oxford Nanopore long reads were assembled into contigs via NextDenovo v2.5.0 (https://github.com/Nextomics/NextDenovo). Mitochondrial contigs were identified by the BLASTn program (Chen et al., 2015) with *Fallopia multiflora* (accession number: MF611850, MF611851) mitogenome as references. And the self-loop candidate contigs were found and polished by Pilon v1.23 (Walker et al., 2014) using Illumina Novaseq sequencing reads, which has been used to assemble the *R. japonica* chloroplast genome before (Chen et al., 2022). Finally, one circular structure of the *R. japonica* mitogenome was obtained. The self-loop mitogenome of *R. japonica* was annotated via online tool GeSeq (Tillich et al., 2017) with the mitogenome of *F. multiflora* (accession number: MF611850, MF611851), and the preliminary annotation was further redressed with the mitogenome of *Fallopia auberti*i (accession number: MW664926). In order to test the credibility of the mitogenome, BWA 0.7.17-r1188 (Li and Durbin, 2009) and samtools v1.9 (Danecek et al., 2021) were used to calculate the sequencing depth of each locus. Finally, the mitogenome map of *R. japonica* was drawn using OGDRAW (Greiner et al., 2019).

The dispersed repeat sequences were analyzed by the online REPuter software (https://bibiserv.cebitec.uni-bielefeld.de/reputer) with the parameter of minimal repeats set to 50 bp, and hamming Distance to 3 (Kurtz et al., 2001). The repeat sequences in the *R. japonica* mitogenome were visualized via Circos v0.69-8 (Krzywinski et al., 2009). The relative synonymous codon usage (RSCU) of the unique protein coding genes (PCGs) of *R. japonica* mitogenome was calculated by CodonW v1.4.4. For the RNA editing sites analysis of the unique PCGs, the RNA-seq data released by our laboratory (PRJNA626400 and RPJNA623335) in the early stage were first filtered via fastp v0.23.2 (Chen et al., 2018), and then mapped to the PCGs of *R. japonica* mitogenome via Bowtie2 v2.3.5.1 (Langmead and Salzberg, 2012). And the possible RNA editing sites were identified via bcftools v1.9 (Danecek et al., 2021) according to the mapping results, and the locations with a coverage depth of more than 10× were selected.

### 2.3 Plastid-like sequences in the mitogenome

The *R. japonica* mitogenome was compared with its chloroplast genome (GenBank accession number: OP583946) by BLASTn with E value less that 1e-5, and visualized via Circos v0.69-8 (Krzywinski et al., 2009).

### 2.4 Selection pressure analysis of PCGs

The PCGs were selected to estimate the selection pressure during the evolution of *R. japonica*. Nonsynonymous (Ka) and synonymous (Ks) substitution rates of the 25 unique PCGs were calculated for *R. japonica* and other three species (*Polygonum aviculare*, *Fallopia aubertii*, and *Fallopia multiflora*). ParaAT2.0 was used to align and format the PCGs with default parameters (Zhang et al., 2012). The Ka, Ks, and Ka/Ks values were calculated via KaKs_Calculator v3.0 following the YN method (Zhang, 2022).

### 2.5 Phylogenetic analysis

The conserved mitogenomes PCGs of *R. japonica* and other ten plant species were identified by BLAT (Kent, 2002). The ten plants, including *F. multiflora* (MF611850 and MF611851), *F. aubertii* (MW664926), *P. aviculare* (OW204033), *Nepenthes X ventrata* (MH798871), *Chenopodium quinoa* (MK182703), *Beta vulgaris* (NC_002511), *Silene vulgaris* (JF750427), *Arachis hypogaea* (MW448460), *Vitis vinifera* (NC_012119), and *Arabidopsis thaliana* (NC_037304) were downloaded from the NCBI. Twelve PCGs (*atp8*, *atp9*, *cox1*, *cox2*, *nad1*, *nad3*, *nad4*, *nad4L*, *nad5*, *nad6*, *nad7*, and *nad9*) were aligned by MAFFT v7.520 (Katoh and Standley, 2013). And GTR + I + G4 model was selected by ModelTest-NG (Darriba et al., 2020) according to Bayesian information criterion scores. Then maximum likelihood (ML) phylogenetic tree was constructed by RAxML-NG v0.9.0 (Kozlov et al., 2019) with 1000 bootstrap replicates.

## 3 Results and discussion

### 3.1 Mitogenome assembly and genomic features

**FIGURE 1:**
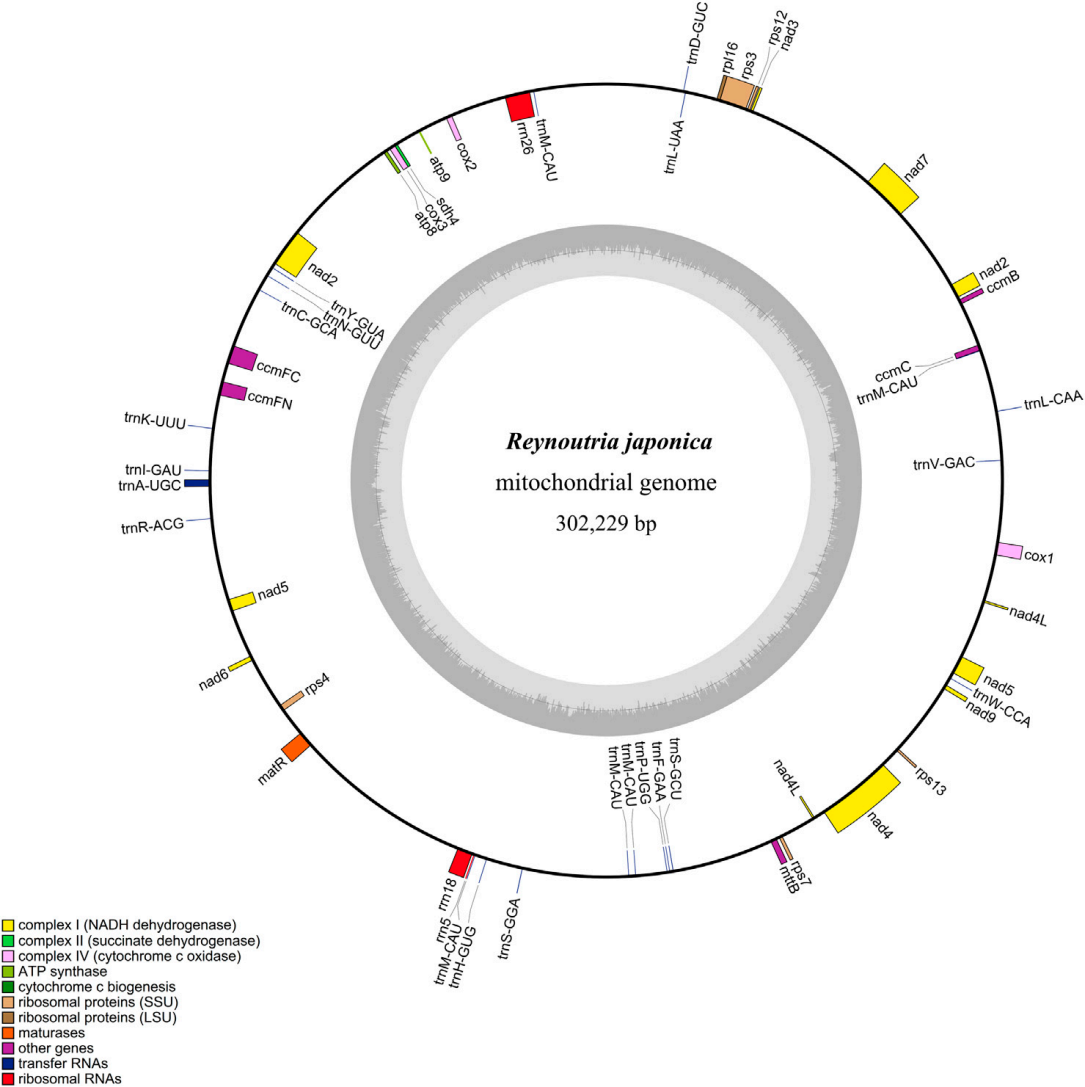
Circular map of the *R. japonica* mitogenome.

The *R. japonica* mitochondrial genome (mitogenome) was first assembled with Oxford Nanopore reads, and then polished with Illumina reads due to the short reads possessing higher base recognition accuracy than long-read sequencing (Delahaye and Nicolas, 2021). This is a common strategy when combining short reads and long reads. By this way, 112 contigs were assembled via NextDenovo. And a self-loop contig was obtained with length of 335,479 bp that can be mapped with the mitogenome of *F. multiflora*. To detect whether it is a circular one, the alignment against itself was performed. Surprisingly, a large fragment was found at the beginning and end, with 99.10% similarity (Supplementary Figure S1) which verifies its circular nature. By removing the tail almost identical sequence, a finally circular structure with 302,229 bp in size was obtained and submitted to NCBI under accession OR228435 (Figure 1). And the average depth was 253× (long reads) and 570× (short reads), respectively (Supplementary Figure S2). The depth of long reads ranging from 3× to 5,212×, achieved all sites of the *R. japonica* mitogenome and made up for the shortcomings of Illumina reads (Supplementary Table S1; Supplementary Figure S2), indicating that the gap-free *R. japonica* mitogenome was obtained. In addition, other 4 contigs, including 3 linear and 1 loop molecular, were also mapped to the *F. multiflora* mitogenome. However, the 3 linear contigs failed to be annotated as mitogenome, and the loop contig was more like a plastid genome than a mitogenome (Supplementary Figure S3).

The base composition of the *R. japonica* mitogenome was A (27.80%), T (27.63%), G (22.32%), and C (22.25%). The mitogenome contained 53 genes, representing 48 unique genes, including 27 protein-coding genes (PCGs), 18 tRNA genes, and 3 rRNA genes (Table 1). The 27 PCGs consisted of 9 NADH dehydrogenase genes (*nad1*, *nad2*, *nad3*, *nad4*, *nad4L*, *nad5*, *nad6*, *nad7*, and *nad9*), a transport membrane protein (*sdh4*), 3 cytochrome C oxidase genes (*cox1*, *cox2*, and *cox3*), 2 ATP synthase genes (*atp8* and *atp9*), a maturase (*matR*), 4 cytochrome C biogenesis genes (*ccmB*, *ccmC*, *ccmFc*, and *ccmFn*), a large ribosomal protein (LSU) gene (*rpl16*) and 5 small ribosomal proteins (SSU) genes (*rps3*, *rps4*, *rps7*, *rps12*, and *rps13*). The total length of these 27 PCGs was 24,678 bp, accounting for 8.17% of the *R. japonica* mitogenome. And the length of tRNA and rRNA genes accounted for 0.56% and 1.71%, respectively. The intergenic region reached a proportion of 89.47%. In the *R. japonica* mitogenome PCGs, only *nad4L* had two copies, and the rest are all single copy. There were 7 unique PCGs containing introns in the *R. japonica* mitogenome (*nad1*, *nad2*, *nad4*, *nad5*, *nad7*, *ccmFC*, and *rps3*). Additionally, three *trans*-spliced introns were found in *nad1*, *nad2*, and *nad5.* Four genes, namely, *mttB*, *rpl16*, *nad1*, and *nad4L* were observed with RNA editing in start codon. A total of 22 tRNAs represented by 18 tRNAs were found, specifying 16 amino acids. And *trnL* and *trnS* possessed two copies, while *trnM* contained five copies (Table 1). By performing the syntenic regions between *R. japonica* and *F. multiflora*, the final mitogenome of *R. japonica* appeared of high similarity to *F. multiflora* (Supplementary Figure S4). Compared with the mitogenome of *F. multiflora*, the *R. japonica* mitogenome has lost several PCGs (namely, *atp1*, *atp4*, *atp6*, *cob1*, *rpl5*, *rps1*, and *rps14*). It was reported that some mitochondrial genes were lost or transferred to the nucleus during the evolution (Adams et al., 2002). The PCGs failed to be screened in the *R. japonica* mitogenome and the 112 assembly contigs might be either lost during the evolution or transferred to the nuclear genome as its nuclear genome is not published yet.

**TABLE 1:** Gene composition of the *R. japonica* mitogenome.

Group of genes	Name of genes
Complex I (NADH dehydrogenase)	*nad1* ^a^, *nad2* ^a^, *nad3*, *nad4* ^a^, *nad4L* (2), *nad5* ^a^, *nad6*, *nad7* ^a^, *nad9*
Complex II (succinate dehydrogenase)	*sdh4*
Complex IV (cytochrome c oxidase)	*cox1*, *cox2*, *cox3*
ATP synthase	*atp8*, *atp9*
Maturases	*matR*
Cytochrome c biogenesis	*ccmB*, *ccmC*, *ccmFC* ^a^, *ccmFN*
Ribosomal protein	*rpl16*, *rps3* ^a^, *rps4*, *rps7*, *rps12*, *rps13*
Transport membrane protein	*mttB*
Ribosomal RNAs	*rrn5*, *rrn18*, *rrn26*
Transfer RNAs	*trnV-GAC*, *trnL-CAA*, *trnM-CAU*(5), *trnL-UAA*, *trnD-GUC*, *trnY-GUA*, *trnN-GUU*, *trnC-GCA*, *trnK-UUU*, *trnI-GAU*, *trnA-UGC*, *trnR-ACG*, *trnH-GUG*, *trnS-GGA*, *trnP-UGG*, *trnF-GAA*, *trnS-GCU*, *trnW-CCA*

^a^ Labeled intron containing genes, and bracketed numbers represent copy number of each gene.

### 3.2 Repeat sequence analysis of *R. japonica* mitogenome

**FIGURE 2:**
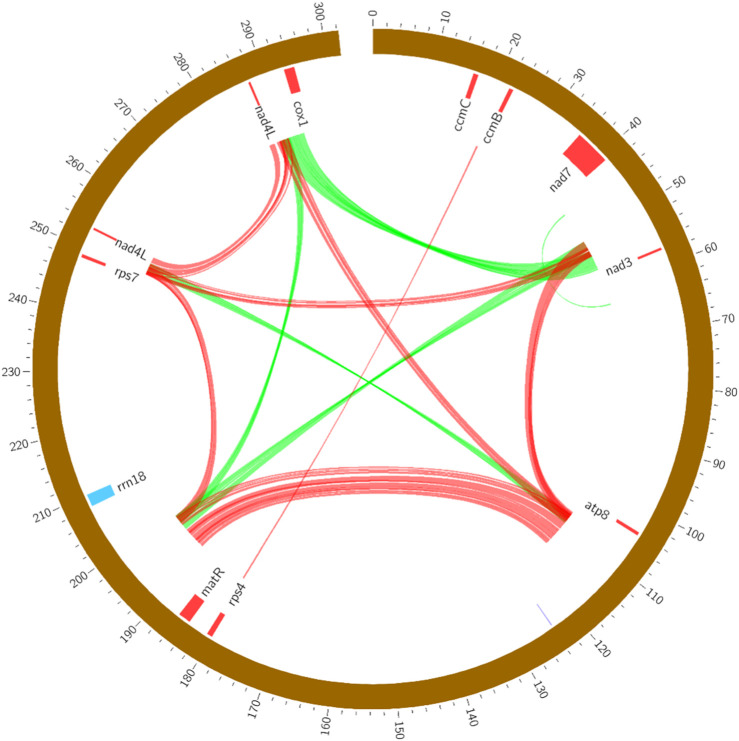
The forward (green) and palindromic (red) repeats in the *R. japonica* mitogenome.

Repeat sequences are the core factor result in the size expansion of plant mitogenome (Alverson et al., 2010). In this study, a total of 54 pairs of repetitive sequences were identified, ranging from 193 bp to 1,983 bp in the *R. japonica* mitogenome (Supplementary Table S2). Seven large repeat sequences were found bigger than 1 kb, which may participate in intramolecular recombination (Bi et al., 2016). It was reported that the repeat fragments could mediate the homologous recombination in the plant mitogenome, such as sweet potato (Yang et al., 2022) and *Scutellaria tsinyunensis* (Li et al., 2021). And the repeats in *R. japonica* mitogenome may also form multiple circular molecules. All the repeats were present primarily as forward or palindromic repeats, and we showed this with different color lines in *R. japonica* mitogenome. As shown in Figure 2, most of the repeat sequences were located in intergenic region, and mainly between *nad7*/*nad3*, *atp8*/*trnY-GUA*, *matR*/*rrn18*, *rps7*/*nad4L*, and *nad4L*/*cox1*. The repeat sequences in the *R. japonica* did not involve any gene, indicating the genes in the *R. japonica* mitogenome are very conservative.

### 3.3 RSCU and RNA editing sites analysis of PCGs

To investigate the codon preferences of PCGs in the *R. japonica* mitogenome, the RSCU analysis was performed. Most PCGs used ATG as the start codon, except *mttB* (ATA), *nad1* (ACG), *nad4L* (ACG), and *rpl16* (GTG). By connecting the 27 unique PCGs with only one start codon (ATG) and a stop codon, a total of 8,174 codons were found in the *R. japonica* mitogenome. Leucine (900, 11.01%) and serine (732, 8.96%) were the two most amino acids, while cystine (124, 1.52%) was the least (Supplementary Table S3). And the most preferentially used codons in the *R. japonica* mitogenome were A-ended or U-ended codons that have RSCU values greater than 1, being consistent with *Hemerocallis citrina* (Zhang et al., 2022), with the exception of threonine (ACC) and leucine (UUG) (Supplementary Figure S5; Supplementary Table S3).

**FIGURE 3:**
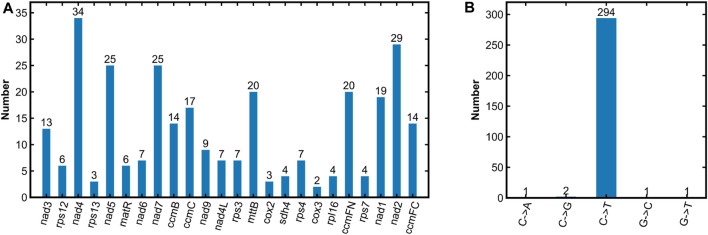
Prediction of RNA editing sites in the *R. japonica* mitogenome. **(A)** Number of RNA editing sites in PCGs. **(B)** RNA editing types and their numbers identified in the *R. japonica* mitogenome.

RNA editing events are widespread phenomenon in plant mitogenome, and have significant impacts on the changes in amino acids (Maier et al., 1996; Grewe et al., 2009). In this study, 299 RNA editing sites were found in the *R. japonica* mitogenome (Supplementary Table S4). Briefly, except for the 3 PCGs (*atp8*, *atp9*, and *cox1*), the remaining 24 PCGs possessed RNA editing sites. And *nad4* had the most RNA editing sites, followed by *nad2*, *nad5*, and *nad7* with 34, 29, 25 and 25, respectively (Figure 3A). Of the five types of RNA editing in *R. japonica* mitogenome, C to T editing had the highest number of occurrences (294 times, 98.33%) and exited in all the 24 PCGs (Figure 3B). C to A editing was only found in *rps4*, and C to G editing was found in both *nad4* and *nad5*. In addition to C to T editing, *nad4* also had C to G, G to T, and G to C editing. As the center of plant energy metabolism, mitochondrion is known as the power house. Base substitution in the sequence of editing key genes may affect plant growth and metabolism. The development of base editor in plant mitogenome (Nakazato et al., 2022) and the mechanism of plant mitogenome editing (Wang et al., 2023b) will help to uncover the mystery of plant mitogenome. The assembly and annotation of *R. japonica* mitogenome will establish a solid foundation in the field about mitochondrial function research.

### 3.4 Horizontal transfer of sequences from the chloroplast genome

**FIGURE 4:**
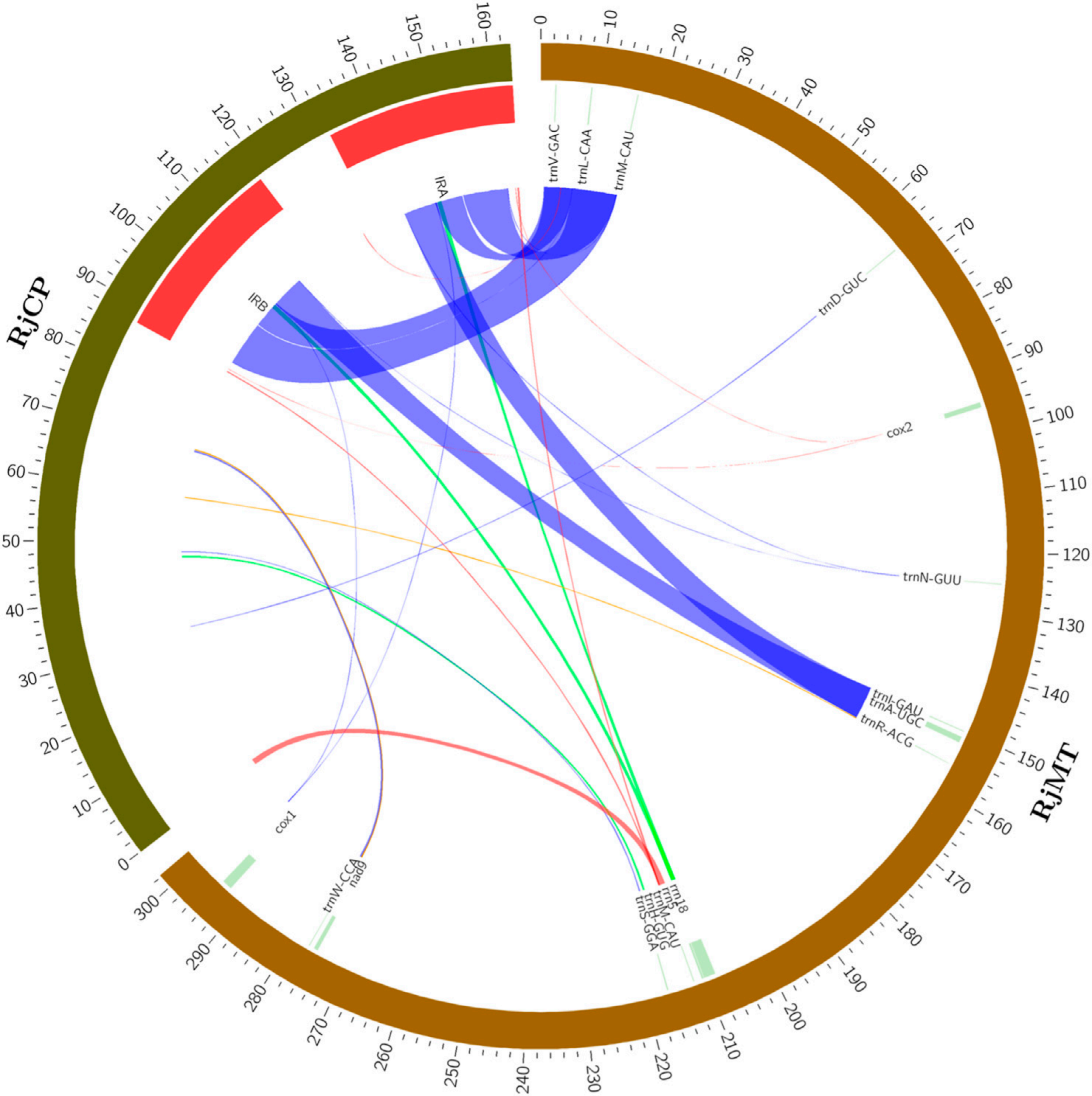
Plastid-like sequences in the *R. japonica* mitogenome. RjCP: the chloroplast genome of *R. japonica*. RjMT: the mitogenome of *R. japonica*. The red arcs represent 100% similarity, the blue arcs represent the similarity between 90% and 100%, the orange arcs represent the similarity between 80% and 90%, and the green arcs represent the similarity between 70% and 80%.

The phenomenon of plant mitogenome containing plastid-like fragments is common in most plant species. It was reported that 23,368 bp in the *Salix wilsonii* mitogenome (accounting for 3.28%) were derived from the chloroplast genome (Han et al., 2022). A total of 21,542 bp in the *Abelmoschus esculentus* mitogenome (accounting for 4.07%) were homologous with its chloroplast genome (Li et al., 2022). In the present study, the plastid-like sequences in the *R. japonica* mitogenome were identified. A total of 17 DNA fragments with a total length of 26,123 bp were similar to chloroplast genome (Supplementary Table S5), accounting for 8.64% of the *R. japonica* mitogenome. Specifically, the plastid-like fragments ranged from 36 bp to 9,402 bp, containing 11 complete tRNA genes (*trnV-GAC*, *trnL-CAA*, *trnM-CAU*, *trnD-GUC*, *trnN-GUU*, *trnI-GAU*, *trnA-UGC*, *trnR-ACG*, *trnH-GUG*, *trnS-GGA*, and *trnW-CCA*) (Figure 4). Additionally, our results demonstrated that the largest plastid-like fragments in the *R. japonica* mitogenome were derived from the inverted repeat region (IRA and IRB, Figure 4) in chloroplast genome. Apart from tRNA genes, the plastid-like sequences mainly located in non-functional fragments which consistent with most other land plants (Straub et al., 2013). The *rrn18* gene in the *R. japonica* mitogenome may migrate from chloroplast genome due to the partial similarity in sequence, and undergo some integration during the evolutionary process.

### 3.5 Selection pressure analyses of mitochondrial PCGs

**FIGURE 5:**
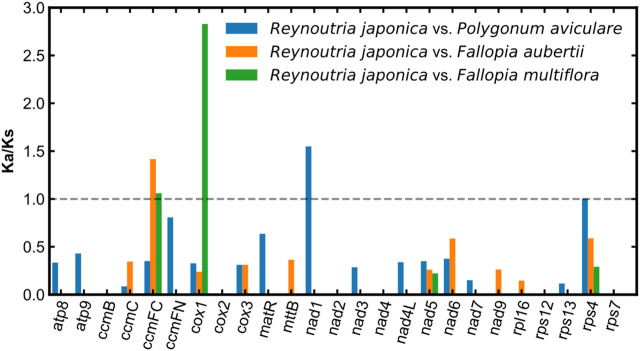
Ka/Ks ratio analysis of the *R. japonica* mitogenome.

In order to estimate the selection pressure of *R. japonica* mitochondrial PCGs, a total of 25 shared PCGs were employed to compute the Ka/Ks ratios among the mitogenome of *R. japonica*, *P. aviculaare*, *F. aubertii*, and *F. multiflora*. The most of the pairwise Ka/Ks ratios were smaller than 1 (Figure 5), indicating that most PCGs were under purifying selection during the evolution of *R. japonica.* And they may play important roles in stabilizing and maintaining the essential function of mitogenome. However, *ccmFC*, *cox1*, and *nad1* were found with Ka/Ks ratios bigger than 1, suggesting that these three genes were subject to positive selection during evolution. Note also that the *cox1* gene had an extremely high Ka/Ks ratio (*R. japonica* vs. *F. multiflora*: 2.83), indicating strong positive selection during the evolution of *R. japonica* and *F. multiflora*.

### 3.6 Phylogenetic analysis

**FIGURE 6:**
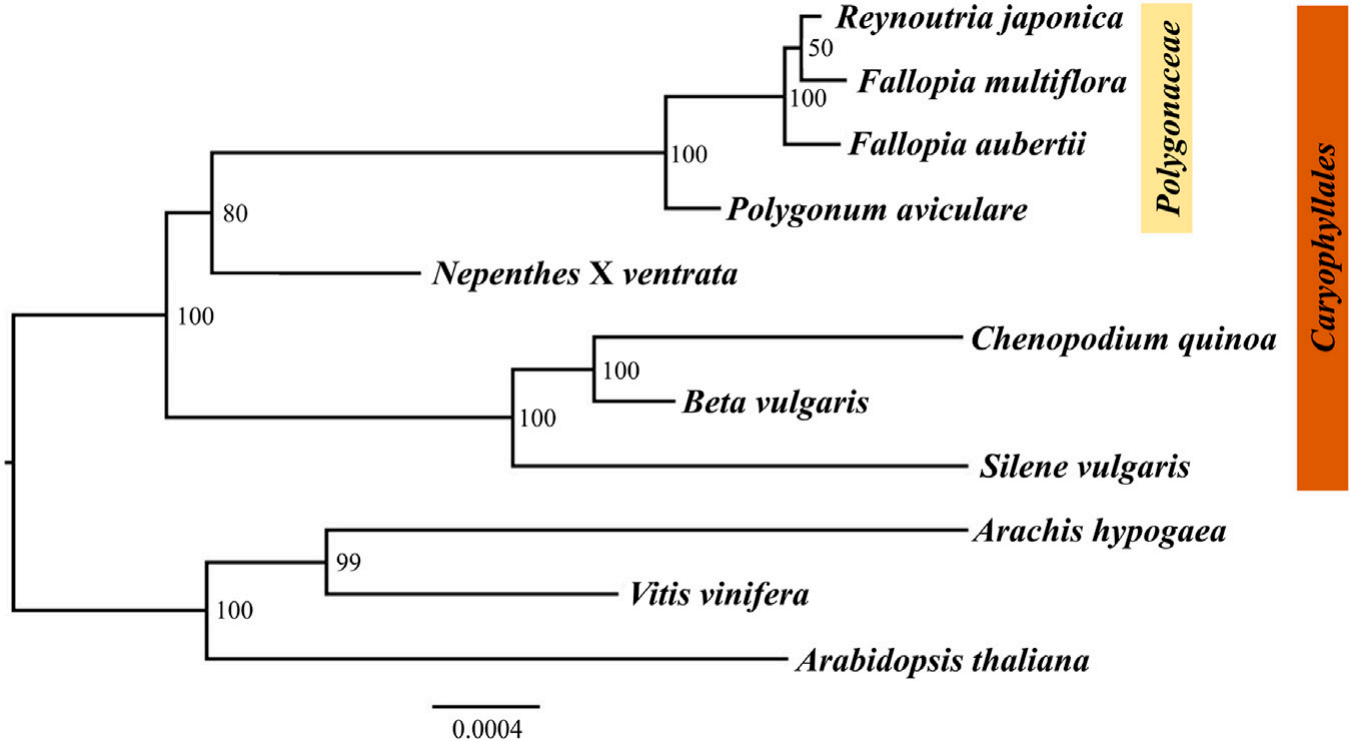
Phylogenetic tree based on twelve homologous protein-coding genes in eleven plants’ mitogenomes using ML analysis. Numbers near the nodes are support values with ML bootstrap values.

The mitogenome is an important tool for developing phylogenetic research. Due to the extensive variations in different plants, the shared conserved PCGs are usually used to conduct phylogenetic analysis. In this study, the maximum likelihood (ML) method was used to construct the phylogenetic tree based on the twelve homologous mitogenome PCGs from eleven species. The results showed that the position of *R. japonica* stayed closest to *F. multiflora*, which is consistent with those based on chloroplast genome (Chen et al., 2022). As shown in Figure 6, the ML tree was divided into two clades, one belongs to the order *Caryophyllales* and others as outgroup. The species in the family *Polygonaceae* (*R. japonica*, *F. multiflora*, *F. aubertii*, and *P. aviculare*) were separated from the other families in the order Caryophyllales, indicating that the mitogenome genes are reliable. The low bootstrap value of *R. japonica* and *F. multiflora* may be due to the high similarity of the mitogenomes (Supplementary Figure S4), indicating a close kinship. And the *R. japonica* mitogenome could provide a kind of reference for further phylogenetic studies.

## 4 Conclusion

This work presented the first mitogenome assembly and annotation of *R. japonica*. The main results include: The *R. japonica* mitogenome was 302,229 bp in length and encoded 48 unique genes, including 27 PCGs, 18 tRNA genes, and 3 rRNA genes. In total, 8,174 codons were encoding the PCGs in the *R. japonica* mitogenome. The noncoding sequences accounted for 89.47% of the *R. japonica* mitogenome where the repeat sequences mainly located. In addition, 11 plastid-like tRNA genes were identified in the *R. japonica* mitogenome, and almost all PCGs were subject to purification selection, except for *ccmFC*, *cox1* and *nad1* which were subject to positive selection. In a word, the current study provided valuable genomic resources for further understanding and utilizing *R. japonica* in the future.

## Data Availability

The datasets presented in this study can be found in online repositories. The names of the repository/repositories and accession number(s) can be found in the article/Supplementary Material.
