# Supplementary material for: De novo assembly and comparative analysis of the mitochondrial genome of Reynoutria japonica
Source: Front Genet. 2023 Nov 23;14:1289811. doi: 10.3389/fgene.2023.1289811 (PMC10702355; doi:10.3389/fgene.2023.1289811)
Supplement: Supplementary file 2 [file DataSheet1.docx]

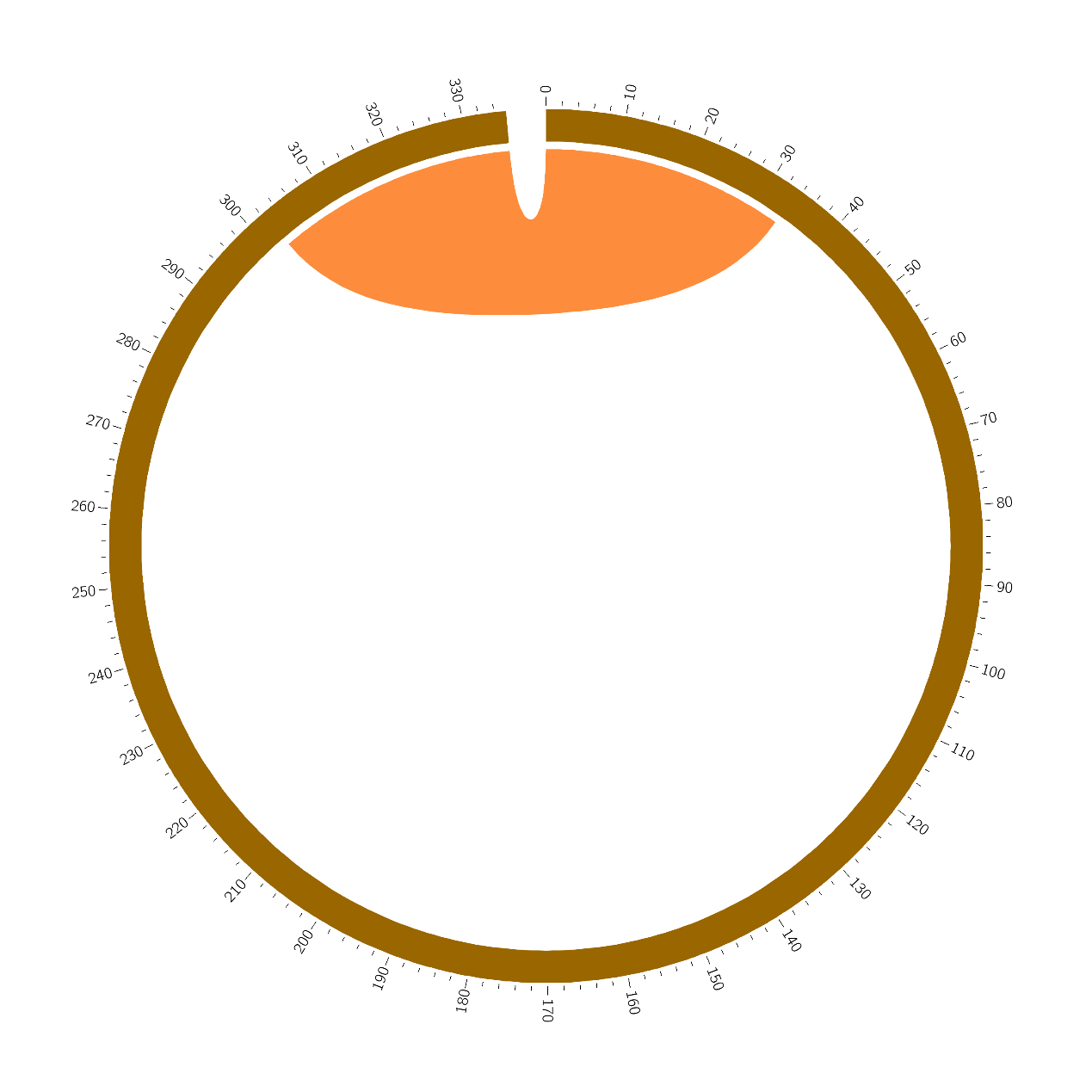


Fig S1. The similarity tail and head fragments in the *R. japonica* mitogenome


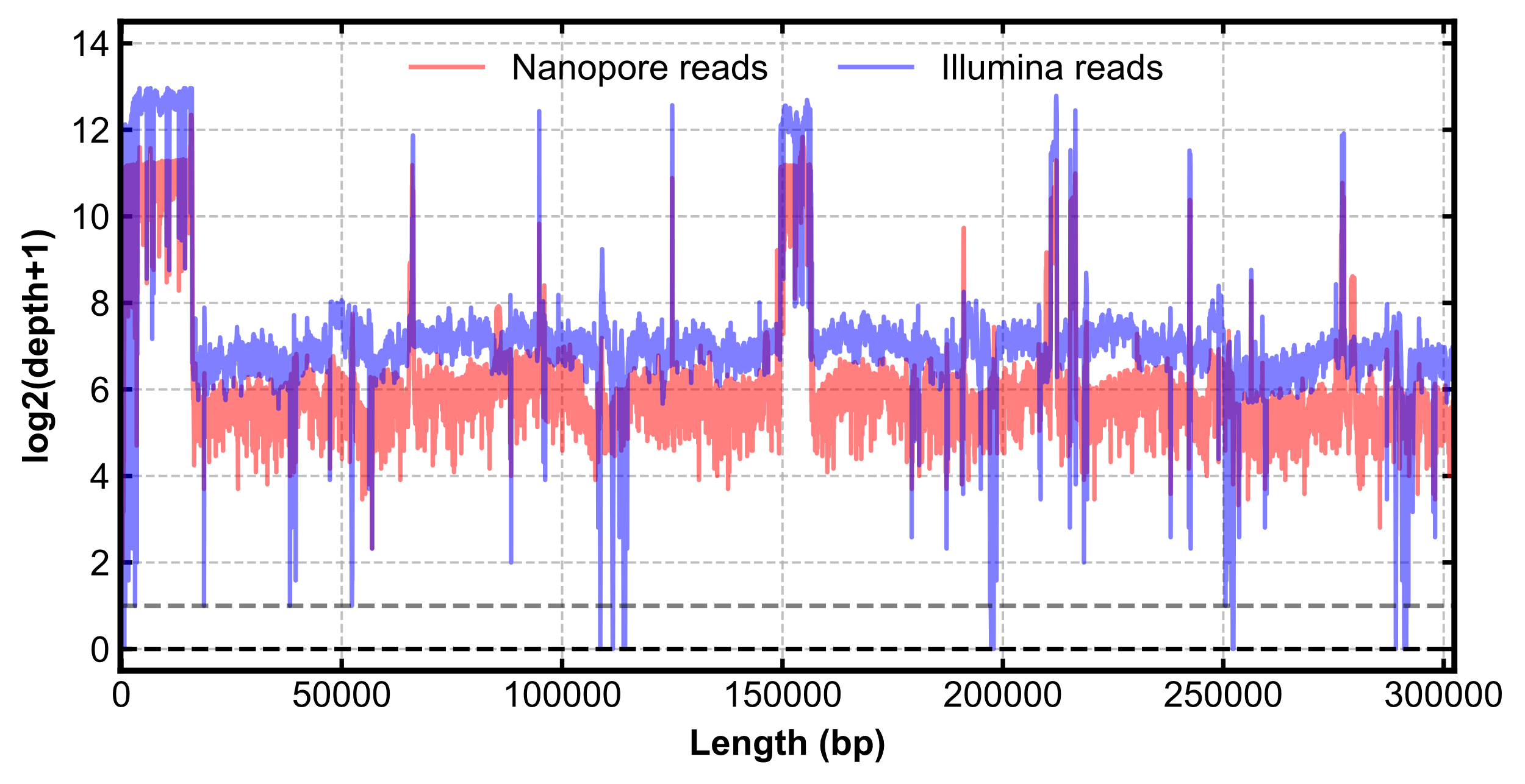


Fig S2. The coverage depth of Nanopore reads and Illumina reads


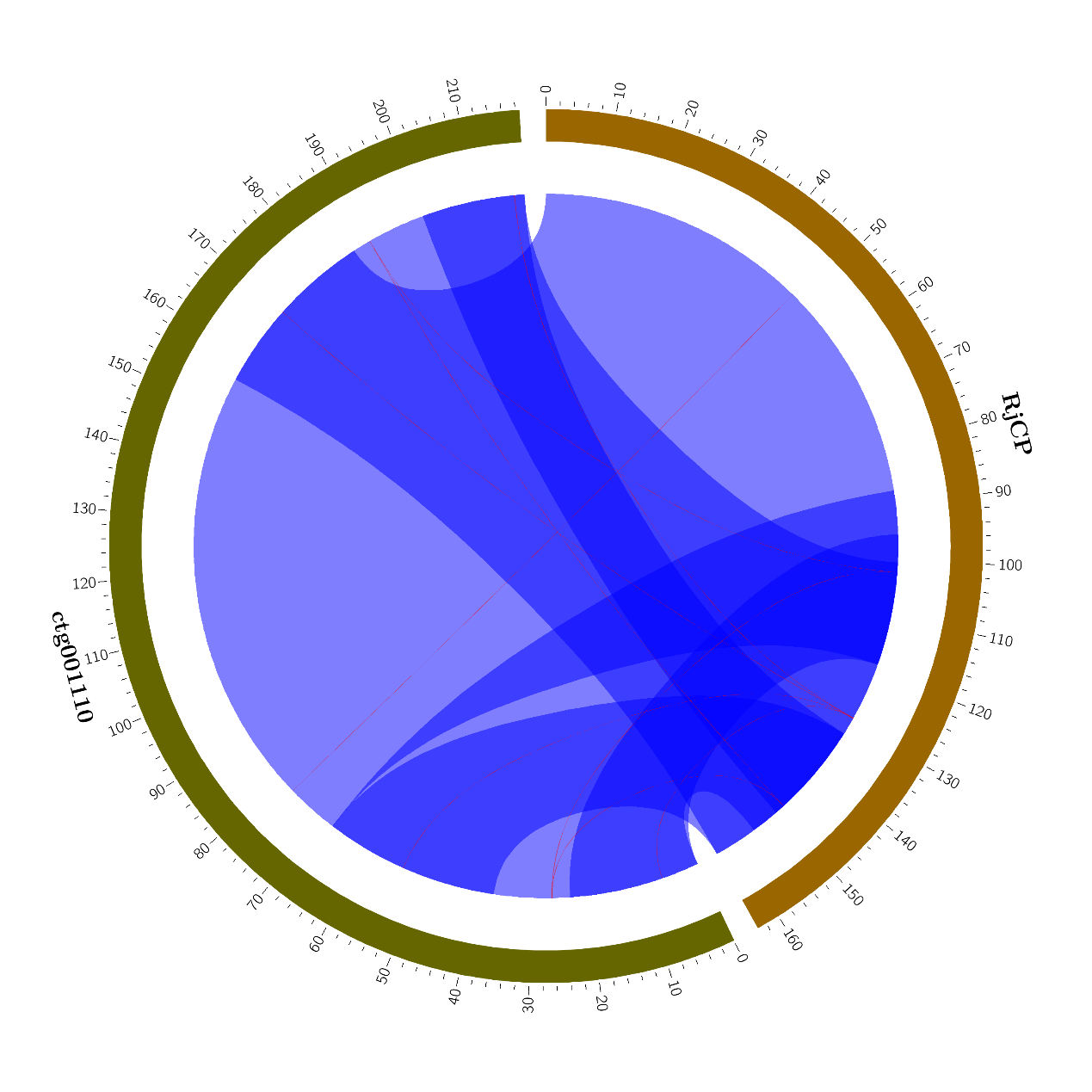


Fig S3. The collinearity between ctg001110 and the chloroplast genome of *R. japonica*

**
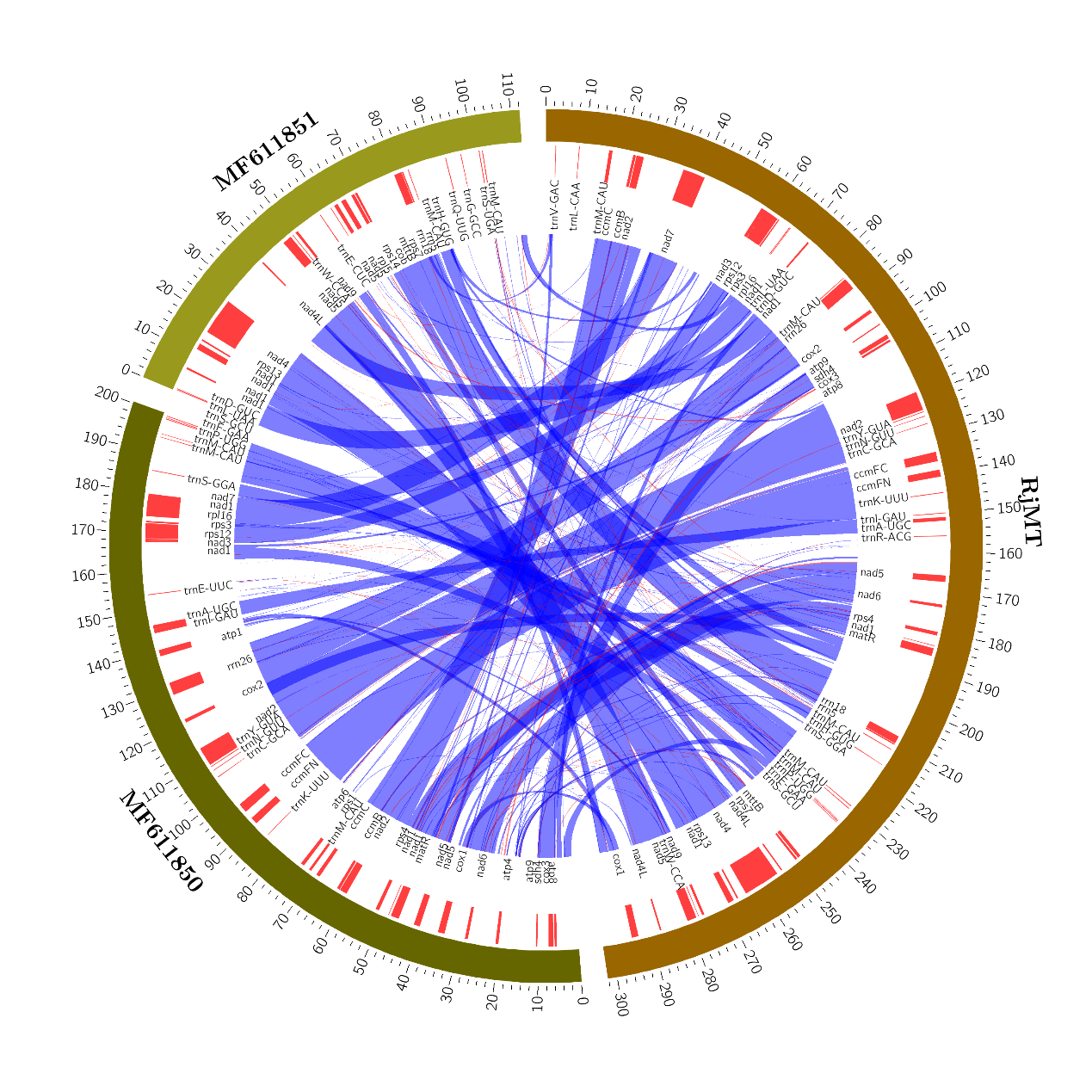
**

**Fig. S4** The mitogenomes syntenic regions between *R. japonica* and *F. multiflora*. RjMT: the mitogenome of *R. japonica*. MF611850 and MF611851: the mitogenome of *F. multiflora*. The red arcs represent 100% similarity, the blue arcs represent the similarity between 90 to 100%, the orange arcs represent the similarity between 80 to 90%, and the green arcs represent the similarity between 70 to 80%


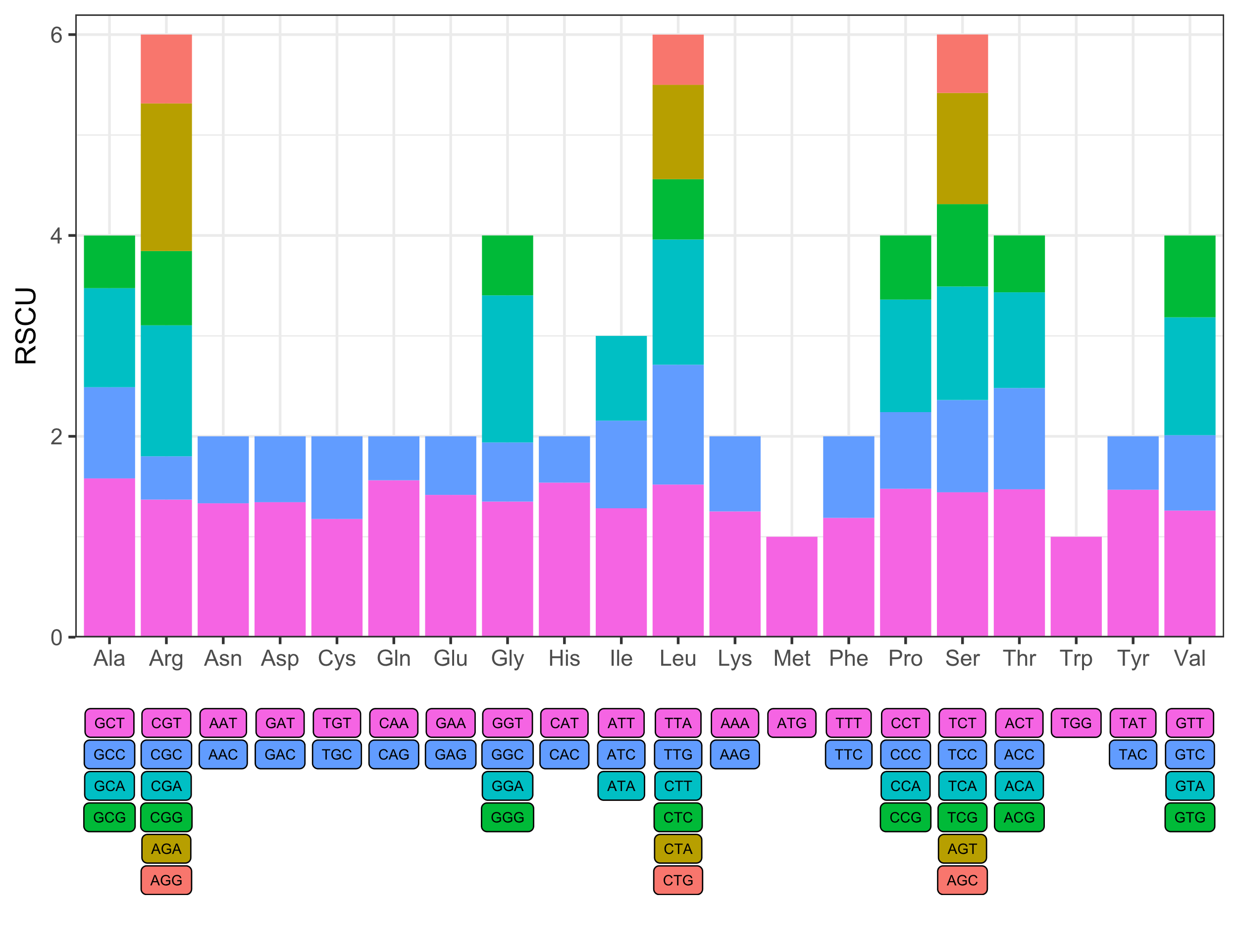


Fig S5. Relative synonymous codon usage (RSCU) analysis of unique PCGs in the *R. japonica* mitogenome.
